# Characteristics of Corn Stover Components Pyrolysis at Low Temperature Based on Detergent Fibers

**DOI:** 10.3389/fbioe.2019.00188

**Published:** 2019-08-02

**Authors:** Fang Wang, Deli Zhang, Mei Chen, Weiming Yi, Lihong Wang

**Affiliations:** ^1^School of Agricultural Engineering and Food Science, Shandong University of Technology, Zibo, China; ^2^Shandong Research Center of Engineering and Technology for Clean Energy, Zibo, China

**Keywords:** corn stover, detergent fibers, low temperature pyrolysis, thermogravimetric analysis, kinetics analysis

## Abstract

In order to study the effect of biomass components on the low temperature (30–400°C) pyrolysis, the thermogravimetric analysis (TGA) of corn stover (CS) and its three detergent fibers (extracted by Van Soest method) were studied, and the model compounds of cellulose, hemicellulose and lignin were also tested as comparison. The results shows the low temperature pyrolysis index (P) is significantly different (P_ADL_ < P_lignin_ < P_CS_ < P_NDF−CS_ < P_xylan_ < P_ADF−CS_ < P_cellulose_). Klason-lignin has stronger thermal stability and decomposes more difficult than alkali lignin. The original cross-linked structure and interaction of the three components inhibited volatiles releasing, especially significant below 300°C. The synergistic effect between cellulose and lignin promoted devolatilization and decreased the initial temperature of cellulose decomposition. At last, the low temperature pyrolysis kinetics parameters (apparent activation energy and pre-exponential factor) of CS and its detergent fibers were calculated via the Coats-Redfern methods. This study can provide a theoretical basis for optimization of process conditions and industrial application of low temperature pyrolysis for lignocellulosic biomass.

## Introduction

China has abundant biomass resource which mainly refers to lignocellulosic materials, including residues and by-products of agriculture, forestry and other related industries (Li et al., 2016). The abundance and renewability of lignocellulosic materials renders them a promising feedstock for cost-effective energy production (Pant et al., 2010). As a thermo-chemical conversion, fast pyrolysis of biomass is not only an process for liquid fuel production that can be used directly, but also an intermediate pretreatment step to convert solid biomass into a higher energy content transportable liquid for subsequent processing for heat, power, biofuels, and chemicals (Bridgwater, 2012). But, the bio-oil obtained by single-step pyrolysis is composed of much complex mixture of oxygenated hydrocarbons with an appreciable proportion of water (Bridgwater, 2012), which leads to a lower calorific value, unstable burning and difficult to refine. The stepwise pyrolysis could improve the utilization of bio-oil according to different thermal decomposition behavior of cellulose, hemicellulose and lignin. Zhang et al. (2016) investigated the two-step pyrolysis of soybean stalk, and the result showed that the bio-oil produced by two-step pyrolysis had significantly higher selectivity toward acetic acid, furfural, methyl catechol, benzene and methylbenzene. Because, as the first step pyrolysis, low temperature pyrolysis would be expected to minimize the moisture content and further decomposition of carbohydrates into smaller oxygenated compounds and produced bio-oil with high calorific value and selectivity (Hammer et al., 2015). In addition, low temperature pyrolysis is also a thermo-chemical pretreatment in several fields of biomass utilization. For example, torrefaction is a mild pyrolysis process at lower temperature used to improve the fuel quality of biomass so that it converts to a more appropriate solid fuel (Acharya et al., 2015). Zhang et al. (2019) compared the effect of dry and wet torrefaction at temperature range from 180°C to 280°C on the chemical characteristic and thermal degradation behavior of corn stalk digestate. Wang et al. (2016) reported a study on lower temperature fast pyrolysis pretreatment that was applied to promote anaerobic digestion efficiency of corn stover.

As a natural polymer, lignocellulosic biomass is mainly composed of cellulose, hemicellulose, and lignin. Pyrolysis characteristics of the three main components are fundamental and essentially important for a better understanding of thermo-chemical conversion of lignocellulosic feedstocks. In recent years, pyrolysis characteristics of lignocellulosic feedstocks and their major components have been extensively studied by many researchers. Many of them investigated the pyrolysis characteristics of cellulose, hemicellulose and lignin using TGA and the pyrolysis temperature ranges of the three main components were obtained (Zhang et al., 2015). However, most of the researches used simplex model compounds rather than complicated lignocellulose to study the pyrolysis characteristics of biomass, which neglected the interaction among the three components and cross-linked structure in biomass.

The detergent fibers are the residues extracted from raw biomass by detergent solution, which can get rid of one component and conserve the other ingredients. The pyrolysis activity of lignocellulosic detergent fibers could not only express the interaction of the components in biomass, but also could provide essential information of the pyrolysis characteristics of individual components by differences. This study used the detergent fibers as model compounds to investigate the low temperature pyrolysis behavior of cellulose, hemicellulose and lignin in biomass, which can evaluate the effect of interaction among the three components and cross-linked structure in biomass.

Therefore, this research studied the low temperature pyrolysis characteristics of CS, which is one of the main agro-residues in the world, using its detergent fibers as model compounds extracted by Van Soest method. In order to investigate the characteristics of each component pyrolysis at low temperature, thermogravimetric analysis and kinetic analysis of CS and its three detergent fibers were studied with the temperature range of 30–400°C, and the model compounds of cellulose, hemicellulose and lignin were also tested as comparison. This study can provide a theoretical basis for low temperature pyrolysis of lignocellulosic biomass, which has a appreciable significance for bio-fuel production with high value and selectivity of chemicals production.

## Materials and Methods

### Raw Materials

CS was collected from Zibo district of Shandong Province, and pulverized to particles with the particle size below 0.147 mm before analysis. The proximate and ultimate analysis of CS are shown in Table 1. The proximate analysis of CS was tested by ASTM standard. The ultimate analysis elemental composition of carbon, hydrogen, nitrogen and sulfur was determined by an elemental analyzer (Vario EL cube, Germany), and the oxygen content was calculated by difference. α-cellulose, xylan and alkali lignin were provided by Sigma corporation (Germany), which were selected as model compounds for cellulose, hemicellulose and lignin.

**Table 1:** Proximate and ultimate analysis of CS.

**Proximate analysis (wt%)**		**Ultimate analysis (dry basis, wt%)**	
Moisture	5.82	Carbon	40.82
Volatile matter	73.53	Hydrogen	5.41
Fixed carbon	15.79	Nitrogen	1.43
Ash	4.39	Sulfur	0.36
		Oxygen (by difference)	47.85

### Detergent Fibers Extraction Experiment

**Figure 1:**
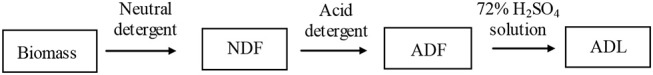
Detergent fiber extraction analysis procedure.

The extraction of detergent fibers was carried out by an extraction unit (FIWE6, VELP Co., Italy), following the analytic procedure developed by Van Soest (Goering and Van, 1971) (Figure 1). The cellulose, hemicellulose, and lignin contents of CS were determined synchronously. NDF is the raw materials removed protein, fat and other extracts, which mainly includes cellulose, hemicellulose, lignin and ash. ADF is the acid detergent fiber that mainly containing cellulose, lignin and ash. And ADL mainly consists of lignin and ash.

### Thermogravimetric Experiment

The pyrolysis characteristics of model compounds of cellulose, xylan, lignin, CS and detergent fibers of CS (NDF, ADF, ADL) were conducted on a TGA (STA449C-QMS403C, Germany). In the TGA experiments, approximately 8 mg sample was heated from 30 to 400°C at a heating rate of 10°C/min, using nitrogen as purge gas with flow rate of 30 mL/min.

### Determination of Reaction Kinetics Parameters

According to the Arrhenius equation and the weight loss under the same heating rate of all samples, the dynamic model expressed as below:

(1) $$\begin{array}{l} \frac{\text{d}\alpha }{\text{d}t}=\frac{A}{\beta }\exp\left(-\frac{E}{RT}\right)f\left(\alpha \right) \end{array}$$

Where α is the conversion ratio of the sample and α = $\frac{\left(M_{0}-M_{t}\right)}{\left(M_{0}-M_{\infty }\right)}$; *t* is time; *M*_0_and *M*_∞_are the initial mass and final mass of the sample, respectively; *M*_*t*_ is the mass of the sample at time t; *A* is the pre-exponential factor; β refers to heating rate of pyrolysis; *E* stands for apparent activation energy; *R* is the universal gas constant; *T* represents the temperature; And f(α) = (1 − α)^*n*^, *n* is the order of reaction.

Integration of Equation (1) according to the Coats and Redfern method gave the following equation (Atul and Rao, 1999):

(2) $$\begin{array}{l} \text{ln}\left[F\left(\alpha \right)\right]=\text{ln}\left[\frac{AR}{\beta E}\left(1-\frac{2RT}{E}\right)\right]-\frac{E}{RT} \end{array}$$

Where,

*F*(α) = ln[-ln(1 − α)/T^2^] for *n* = 1

*F*(α) = ln[1 − (1−α)^(1−*n*)^/T^2^(1 − *n*)] for *n* ≠ 1

For the thermal degradation of biomass, 2RT/E is very small (2RT/E < < 1), so the value of $\text{ln}[\frac{AR}{\beta E}(1-\frac{2RT}{E})$ is approximate to a constant. Therefore, the kinetic parameters (apparent activation energy and pre-exponential factor) could be obtained from the slope and intercept of linear fitting about Equation (2). Different values of n were used to fit and selected according to the linear fitting degree (R^2^).

## Results and Discussion

### Analysis of Detergent Fibers

Table 2 lists the lignocellulosic composition of the raw CS and its detergent fibers. From the relative contents of each component in the detergent fibers, the pyrolysis behavior of NDF-CS samples could reflect the synergy of the three major components. Since cellulose accounts for 79.49% in ADF-CS samples, the pyrolysis characteristics of cellulose dominate the ADF-CS pyrolysis behavior. Lignin and a portion of insoluble ash are the main components in ADL-CS samples, which is also called Klason-lignin (Shen et al., 2015). This study investigated the pyrolysis characteristics of each CS components based on the differences among CS and its detergent fibers.

**Table 2:** Component of corn stover and its detergent fibers (wt.%).

**Samples**	**extractives**	**Hemicellulose**	**Cellulose**	**Lignin**	**Undissolved ash**
CS	16.95	31.39	41.05	6.34	4.27
NDF-CS	–	37.89	49.43	7.62	5.15
ADF-CS	–	–	79.49	12.25	8.28
ADL-CS	–	–	–	59.68	40.31

### Thermogravimetric Analysis of Corn Stover and Its Detergent Fibers

**Figure 2:**
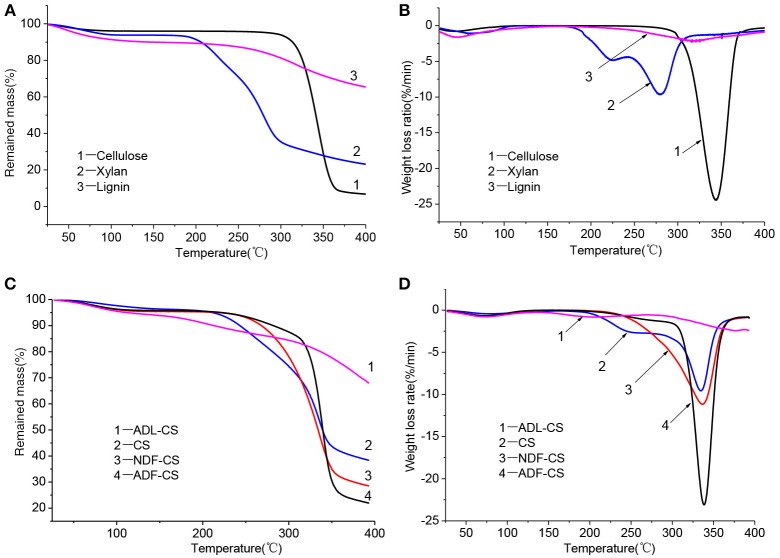
TG and DTG curves for three simplex model compounds (cellulose, xylan and lignin) **(A,B)**, CS and its detergent fibers **(C,D)** at low temperature.

The TGA of the three model compounds (cellulose, xylan and lignin) was studied under nitrogen atmosphere (Figures 2A,B). The pyrolysis characteristics of three samples showed great differences which can be related to the different chemical structures among cellulose, xylan and lignin (Quan et al., 2016). Due to existence of side and branched chains of hemicellulose and lignin, their initial pyrolysis temperature was lower than that of cellulose. While, cellulose showed a sharp derivative thermogravimetric (DTG) peak at 345°C. In the temperature range of 30–400°C, the pyrolysis stability of lignin is much higher than cellulose and xylan. The solid residue left from lignin pyrolysis is 65.42% at 400°C, which illustrates the lignin is still not completely pyrolysis in the temperature range.

Figures 2C,D shows the thermogravimetric (TG) and DTG curves of CS and its detergent fibers pyrolysis at low temperature. The thermal weight loss of four samples could be approximately divided into three stages with temperature range of 30–400°C according to peaks of DTG curves, including moisture evaporation and slight degradation step, main pyrolysis step and carbonization step. In the first step, the moisture was removed and some low molecular weight products generated. With the increase of temperature, large quantity of volatile released in main pyrolysis step due to the cleavage of main chain. Carbonization step had a slight weight loss with a small temperature range. The weight loss ratio of CS and its detergent fibers in different pyrolysis stage is showed in Table 3. As shown in Figure 2D, a small peak appeared in the initial stage of the four samples pyrolysis caused by moisture evaporation, and in the ADL-CS samples, the reduction of free water was more severe because of absorption of moisture determined by lignin (Chen et al., 2012). When the pyrolysis temperature reached to around 150°C, all the samples turned to slight degradation step, and partly depolymerization and glass transition phenomena appeared. But, the initial temperature that volatile matters of the four samples released was different (T_ADL−CS_ < T_CS_ < T_NDF−CS_ < T_ADF−CS_), which is determined by the constituents of CS and its detergent fibers. The order is proportional to the proportion of cellulose and inversely proportional to the content of lignin in the samples. All the samples appeared a weight loss peak in the main pyrolysis step except ADL-CS. The DTG curve of the CS had a shoulder peak at around 225°C, which is believed the peak resulting from hemicellulose pyrolysis. A high proportion of hemicellulose in samples will result in an obvious shoulder peak in DTG curve (George et al., 2006). But, the shoulder peak disappeared in the DTG curve of NDF-CS pyrolysis, instead of a single wider peak, which may be a result of reduction of neutral extractives leading to postponing temperature of hemicellulose pyrolysis. Guo et al. (2010) research indicated neutral extractives can promote production of carboxylic acids, which is the main production of hemicellulose in the lower temperature pyrolysis (Lv et al., 2010). The DTG curve of ADF-CS had a sharp peak at around 337°C which reflecting the pyrolysis characteristics of cellulose. The DTG curve of ADL-CS appeared two slight peaks. The first one is the result of cleavage of side chains in lignin and generation of smaller molecules including CO, CO_2_ and H_2_O etc. The second one is caused by degradation of main chain in lignin, which was weakly in the temperature range. In addition, the corresponding temperature of maximum weight loss peak of CS, NDF-CS and ADF-CS (334–338°C) was similar to cellulose. The result showed there was no significant influence on the corresponding temperature of maximum pyrolysis peak by changing the composition of extractives, hemicellulose and lignin within a certain range.

**Table 3:** Weight loss ratio of CS and its detergent fibers in different pyrolysis stage (30–400°C).

**Sample**	**Drying and slight degradation step**		**Main pyrolysis step**		**Carbonization step**	
	**Temperature range (°C)**	**Weight loss ratio (%)**	**Temperature range (°C)**	**Weight loss ratio (%)**	**Temperature range (°C)**	**Weight loss ratio (%)**
CS	30–210	4.94	210–382	55.75	382–400	1.01
NDF-CS	30–235	5.12	235–388	65.48	388–400	1.23
ADF-CS	30–245	5.96	245–375	70.40	375–400	1.09
ADL-CS	30–170	6.95	170–400	26.10	–	–

The solid residue yield (S) of four samples at 400°C was significant different (S_ADF−CS_ < S_NDF−CS_ < S_CS_ < S_ADL−SC_). The differences of cellulose and lignin pyrolysis characteristics resulted in the highest value of S for ADL-CS, while ADF-CS was the lowest. The char yield of NDF-CS pyrolysis was significantly less than CS, which could be attributed to the reduction of extractives. The extractives can promote the generation of coke during CS pyrolysis (Raveendran et al., 1996). whose one reason is a portion of ash dissolved in neutral detergent, and another reason is the reduction of extractives, because the extractives can promote the generation of coke during CS pyrolysis (Raveendran et al., 1996).

Pyrolysis parameters of all samples could be obtained by TG and DTG curves, as shown in Table 4. However, the direction of the parameters is dispersed that cannot evaluate the pyrolysis characteristics comprehensively. So a low temperature pyrolysis index (*P*) was used to describe devolatilization characteristics of samples at lower temperature based on pyrolysis parameters developing from the method of Fu et al. (2009), as shown in Equation (3).

(3) $$\begin{array}{l} P=\frac{D_{\text{max}}\;\cdot \;D_{\text{avg}}}{T_{\text{s}}\;\cdot \;T_{\text{max}}\;\cdot \;S} \end{array}$$

Where, *T*_*s*_ is the initial temperature of volatiles releasing; *D*_*max*_ refers to the maximum weight loss rate of volatiles (the peak value of DTG); *T*_*max*_ is the corresponding temperature of D_max_; *D*_*avg*_ represents the average weight loss rate of volatiles; *S* stands for solid residue yield at terminal temperature.

**Table 4:** Pyrolytic parameters of three simplex model compounds (cellulose, xylan and lignin) (a,b), CS and its detergent fibers.

**Sample**	**T_**s**_ (°C)**	**D_max_** ** (%· min^**−1**^)**	**T_**max**_ (°C)**	**D_avg_** **(% · min^**−1**^)**	**S (%)**	**P (10^**−5**^)**
cellulose	296	−24.7	340	−2.33	6.82	8.28
Xylan	185	−10.17	278	−1.92	23.15	1.77
Lignin	170	–.6	320	−0.87	65.44	0.07
CS	205	−9.57	334	−1.54	38.32	0.56
NDF-CS	235	−10.16	336	−1.79	28.46	0.81
ADF-CS	245	−23.1	337	−1.95	21.82	2.50
ADL-CS	170	2.5	375	−0.81	67.80	0.04

A high value of *P* can illustrates that the sample has an excellent performance of volatiles releasing at low temperature pyrolysis, and the order is P_ADL−CS_ < P_lignin_ < P_CS_ < P_NDF−CS_ < P_xylan_ < P_ADF−CS_ < P_cellulose_, which is mainly decided by the diversity of constituents in samples and pyrolysis characteristics of cellulose, hemicellulose and lignin. Although the *T*_*s*_ of NDF-CS increased by reduction of extractives from CS, the *P*_*NDF*−*CS*_ was still higher than P_CS_. After washing by neutral detergent, the structure of NDF-CS became more loose, leading to the decomposition of volatiles is more severe (S_NDF−CS_ < S_CS_). The result of *P*_*ADL*−*CS*_ < *P*_*lignin*_ showed that klason-lignin has stronger thermal stability and decomposes more difficult than alkali lignin, and the same result was concluded by Wang et al. (2015), whose reason is the condensation reaction by H_2_SO_4_ during the preparation of ADL. As shown in their DTG curves (Figures 2B,D), the pyrolysis characteristics of ADL-CS and lignin have significant differences.

### Pyrolysis Characteristic Comparison of Detergent Fibers and Model Compounds of CS

According to the proportion of cellulose, hemicellulose and lignin distributed in NDF-CS and ADF-CS, cumulative calculation of the weight loss of three model compounds deduced TG curves of NDF-CS(cal) and ADF-CS(cal), respectively, following the formula as below:

(4) $$\begin{array}{l} S_{DF}=S_{C}\;\times \;X_{C}\;+\;S_{H}\;\times \;X_{H}\;+\;S_{L}\;\times \;X_{L}\;+\;X_{\text{ash}} \end{array}$$

Where, *S*_*DF*_, *S*_*C*_, *S*_*H*_ and *S*_*L*_ stand for solid residue yield of detergent fiber, cellulose, hemicellulose and lignin; *X*_*C*_, *X*_*H*_, *X*_*L*_ and *X*_*ash*_ are the proportions of cellulose, hemicellulose, lignin and ash distributed in detergent fibers, respectively.

**Figure 3:**
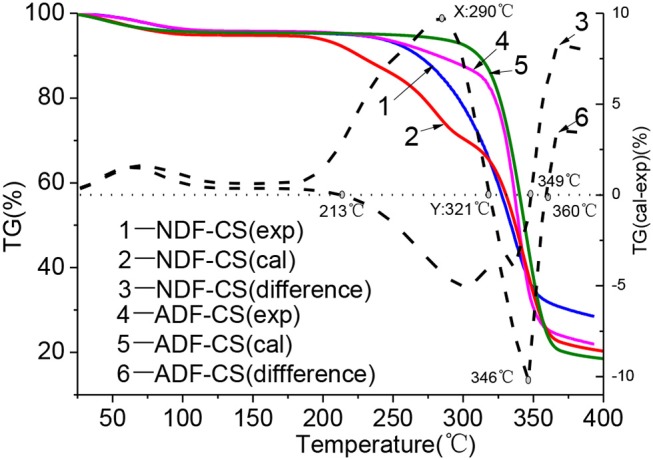
Pyrolysis characteristic differences between experimental and calculated values of NDF-CS and ADF-CS.

The influences of interaction and cross-linked structure in detergent fibers on devolatilization characteristics at low temperature could be obtained by comparison with the differences between experimental and calculated TG values of detergent fibers, as shown the imaginary line in Figure 3. Many studies proved that washing can eliminate the effect of ash on pyrolysis characteristics of biomass by water or weak acids (Jiang et al., 2013), so the TG curves of NDF-CS and ADF-CS can mostly reflect the synergistic pyrolysis characteristics of cellulose, hemicellulose and lignin. Furthermore, the construction of lignin was damaged seriously in ADL after washing, so pyrolysis characteristics of ADL-CS were not given.

As shown in Figure 3, before 200°C, the samples stayed in moisture evaporation and slight degradation step and volatiles releasing slightly, so there is weaker impact on devolatilization by interaction of original component.

With the increase of temperature, the difference between experimental TG value and calculated values became more seriously. The intensity of inhibitory action on pyrolysis and devolatilization was increased by interaction of components in NDF-CS, and it was the strongest when the temperature reach to 290°C (X). Then, intensity of inhibitory action was declined till 321°C (Y). The differences between experimental value and calculated value were negative in the temperature range of 321–349°C, which showed the positive effect on devolatilization. After 349°C, the intensity of inhibitory action was enhanced again. During pyrolysis of NDF-CS, the temperature point of X and Y is nearly identical with the *T*_*s*_ of cellulose (296°C) and the terminal temperature point (325°C) of main pyrolysis step of hemicellulose. As the increase of temperature, hemicellulose decomposed firstly and formed liquid covering the surface of cellulose, which resulted in the inhibition of volatiles releasing from cellulose (Hosoya et al., 2007). But this cannot explain the inhibition among constituents in NDF-CS before 296°C, when the cellulose was relative stable. The phenomenon may be main caused by the original cross-linked structure of CS. Previous studies show that in biomass, the hemicellulose is connected with cellulose and lignin by the hydrogen and covalent bonds, and the bond energy are approximate 50 kJ/mol and 100–400 kJ/mol, respectively (Sofia et al., 2009; Zhang et al., 2011). When the pyrolysis temperature is lower, the bond between hemicellulose and other components inhibits the devolatilization, which was also concluded by Svenson et al. (2004). The TG curves of birch and calculated value of cellulose, hemicellulose and lignin in the temperature range of 300-600°C were compared, and the result showed there was significant difference at 300°C. But little differences in the temperature range of 400–600°C. As the temperature further increased, volatiles releasing of hemicellulose nearly completely, and the degradation of lignin in NDF-CS occurred. The volatile production of cellulose acted as H-donors, and the free radicals generated from lignin degradation acted as H-acceptors, which can promote cellulose decomposing to small molecule gases (Hosoya et al., 2009). The low temperature pyrolysis of cellulose can provide additional free radicals promoted scission of lignin and lignin-derivatives, which can produce a synergistic effect between cellulose and lignin devolatilization. The synergistic effect is beneficial for volatilize of cellulose and lignin.

Compared the differences of TG curves between experimental value and calculated value of ADF-CS, the result showed that in the initial main pyrolysis step, interaction of components promoted the devolatilization of ADF-CS. The promotion was enhanced with the increase of temperature until 346°C. Then, the promotion decreased gradually. In the process of ADF-CS pyrolysis, the devolatilization was advanced before 296°C (similar to the *T*_*s*_ of cellulose). Although the chemical bond connected all the constitutes inhibited volatiles releasing, the distribution of components was more compact and the thermal conductivity of lignin was higher than cellulose, leading to the faster heat transformation of ADF-CS. In addition, the surface of raw biomass still kept fibrous and porous during pyrolysis (Gani and Naruse, 2007) which further enhanced the release of volatiles in ADF-CS. Furthermore, with the temperature increasing, the influence from cross-linked structure in raw biomass became weaker and the synergistic reaction between cellulose and lignin was stronger, which resulted in the interaction of components was much stronger. This can provided a theoretical direction for industrial application of low temperature pyrolysis of original biomass, such as torrefaction.

When the temperature increased to 370°C, the experimental TG curves of NDF-CS and ADF-CS showed inhibiting action, which may be caused by the difference pyrolysis behaviors between alkali lignin and lignin in detergent fibers (Wang et al., 2007). Besides, the dehydration sugar is easy to polymerization to promote carbonization reaction of lignin during pyrolysis (Haruo et al., 2003).

Overall, the inhibition of interactions among the components in NDF-CS was stronger than that in ADF-CS in general, whose main reason was the difference among the components as mentioned above. Meanwhile, the productions of hemicellulose were similar with cellulose during biomass pyrolysis, which further inhibited degradation of cellulose (Collard and Blin, 2014). Moreover, the slight damage of the cross-linked structure cannot be neglected by the acid detergent washing, leading to reducing the inhibition effect of ADF-CS.

### Low Temperature Pyrolysis Kinetic Analysis of CS Components

The low temperature pyrolysis kinetics parameters of CS, NDF-CS, ADF-CS, NDF-CS (cal) and ADF-CS (cal) were calculated via the Coats-Redfern methods, as shown in Table 5. When the value of n was 2, the R^2^ was optimal. The *E* of CS is lower than NDF-CS and ADF-CS, which may be caused by some components removing during process of washing, including neutral extracts and hemicellulose. The *A* of the three samples increases in turn, which is attributed to cellulose relative content raising during process of washing. With the increase of *E* and *A*, the pyrolysis temperature range and the peak value of DTG is more higher, which is corresponding to previous analysis in this paper.

**Table 5:** Pyrolysis kinetics parameters of CS and its detergent fibers.

**Sample**	**Temperature range/°C**	***n***	**E/kJ · mol^**−1**^**	**A/s^**−1**^**	***R*^2^**
CS	300–380	2	53.8	1.7 × 10^8^	0.964
NDF-CS(exp)	300–390	2	84.3	1.845 × 10^15^	0.985
NDF-CS(cal)	310–390	2	75.8	8.512 × 10^11^	0.979
ADF-CS(exp)	300–380	2	112.9	3.602 × 10^19^	0.978
ADF-CS(cal)	300–380	2	100.5	4.312 × 10^15^	0.990

The calculated *E* and *A* of NDF-CS and ADF-CS all are lower than the experimental values, which could be attributed by (1) the original cross-linked structure of NDF-CS and ADF-CS, which lead to the components connect with each other, and more energy to break the original structure. (2) the synergistic effect of cellulose and lignin, which resulted in the increase of *A*.

## Conclusion

The low temperature (30–400°C) pyrolysis characteristics of CS detergent fibers and model components is significantly different by comparison the TG and DTG curves and the low temperature pyrolysis index (*P*). The tendency of TG curves of NDF-CS_(difference)_ and ADF-CS_(difference)_ illustrated that the effect of components interaction and original cross-linked structure in CS on devolatilization characteristics and enhanced the activation energy and pre-exponential factor of samples. When the pyrolysis temperature is below to 200°C, the effect of interaction among three components and cross-linked structure on pyrolysis behavior of biomass can be neglect. While, when the temperature ranges from 200 to 400°C, the effect cannot be neglect. The effect inhibits the release of pyrolysis volatiles in the whole pyrolysis process. But, in the main pyrolysis region of cellulose, the interaction between lignin and cellulose has obvious synergistic effect on volatile releasing and decrease the initial temperature of the cellulose volatilization. In the future work, how the components interaction and original cross-linked structure of lignocellulosic biomass effect the complex pyrolytic reaction and the pyrolysis products should be further study. This study can provide a theoretical basis for choosing a reasonable temperature region and optimization of process conditions according to different purpose of low temperature pyrolysis.

## Data Availability

The datasets generated for this study are available on request to the corresponding author.

## Author Contributions

FW participated in the experiment and writing of text. DZ participated in the analysis of data and writing of text. MC participated in the experiment. WY and LW participated in the theoretical analysis of the research.

### Conflict of Interest Statement

The authors declare that the research was conducted in the absence of any commercial or financial relationships that could be construed as a potential conflict of interest.

## Abbreviations

ADF: Acid detergent fiber
ADL: Acid detergent lignin
CS: Corn stover
DTG: Derivative thermogravimetric
NDF: Neutral detergent fiber
TG: Thermogravimetric
TGA: Thermogravimetric analysis.

## Nomenclature

α Conversion ratio

*M* Mass of sample

*A* Pre-exponential factor

*E* Apparent activation energy β Heating rate of pyrolysis

*R* Universal gas constant

*n* Order of reaction

*T*_*s*_ Initial temperature of volatiles releasing

P Pyrolysis index

*D* Weight loss rate of volatiles

*S* Solid residue yield at terminal temperature

*S*_*DF*_, *S*_*C*_, *S*_*H*_, *S*_*L*_ Solid residue yield of detergent fiber, cellulose, hemicellulose and lignin

*X*_*C*_, *X*_*H*_, *X*_*L*_, *X*_*ash*_ Proportions of cellulose, hemicellulose, lignin and ash distributed in detergent fibers
